# Some Account of the Disease Called by the People “Black Tongue,” Which Prevailed in Some Parts of Missouri, in the Winter of 1842–’3

**Published:** 1843-08

**Authors:** E. H. Bennett

**Affiliations:** New Madrid, Missouri


### Art. VII.—*Some Account of the Disease called by the people "Black Tongue," which prevailed in some parts of Missouri, in the winter of* 1842–'3. By Dr. E. H. Bennett, of New Madrid, Missouri.

This disease, which appeared first in our neighborhood at Point Pleasant, eight miles south of New Madrid, early in the winter, and prevailed with uncommon mortality during the cold season, comes on with a feeling of lassitude, loss of appetite, slow fever, thirst, fulness of head, sneezing, cough, and an acrid watery discharge from the nose and eyes. These symptoms constitute the stage of access, and in violent cases are succeeded in from five to ten hours by a severe chill, and excruciating pains sometimes in the forehead, and again in the eyes, which occasionally in a few hours swell, close, and grow black, as if bruised by a blow. In other cases the pain is felt in the end of the nose, or in the side of the head, in the neck, the shoulder, the elbow joint, the wrist, or some of the fingers. Often the seat of pain is the breast, side, or abdomen, in the region of the liver, heart, stomach, or kidneys, and in other instances the pain is most intense in the lower extremities. The chill in many cases of the epidemic persisted until the patient sunk under the attack; but generally after a cold stage of from nine to twelve hours a partial reaction took place, with dryness of the mouth, parched tongue, a dark stripe in the middle, and white edges. In some of the cases, the tongue was natural, in others too pale, and in these the secretions were natural.—Some complained of a sense of burning in the stomach, and of inextinguishable thirst. The throat, in some instances, was the principal seat of the disease, swelling to such an extent as to render deglutition almost impracticable, and greatly embarrass respiration, the face becoming livid and *œdematous*, the pulse feeble and tremulous, extremities cold, perspiration limited to the face, and cold.

In twenty-four or thirty hours, in most cases, the swelling of the throat subsided, and the breathing and swallowing became free. The pain was then transferred to the region of the heart, extending down both sides to the region of the kidneys, exciting a continual desire of micturition. These symptoms were succeeded by others of greater violence—as cold extremities, incessant tossing, wild countenance, and death, in from ten to twenty-four hours. During the month of January, of this character I had five cases, of which four had a fatal termination. It was this aspect of the endemic which excited the fears of our citizens, that the disease was that which had been reported as prevailing with great mortality in New York, and was called the "*black tongue.*" The tongue becoming deeply engorged by the tumefaction in the throat, coated with a dark brown fur, and protruded, as it occasionally was, beyond the teeth, presented a feature well calculated to give that name to the disease. It was not the usual progress of the complaint, but in some cases a decided re-action succeeded to the symptoms of depression, requiring an active depletory treatment. In far the greater proportion of cases, the type was decidedly typhoid, and that treatment which I found most successful was a combination of the anodyne and stimulant, topical rubefacients having been used extensively, and with marked advantage. As the weather began to grow warmer the disease put on rather more of an inflammatory type, but an active cathartic rarely failed, even then, to convert the case into one of a typhoid character, in which collapse not unfrequently appeared in a few hours, to the destruction of the patient.

In January, I attended one hundred and fourteen cases of the disease, five of which were fatal; in February, I had sixty cases, and lost but one; in March, fifty, losing five; in April, between fifty and sixty, of which I lost two. The following case occurred in the latter month, and is reported as affording a good epitome of the features of the epidemic:

A negro man, forty-eight years of age, of robust frame, was attacked on the night of the 6th of April, with the prevailing disease. For a year previously, he had been troubled with a dry cough, and symptoms of chronic hepatitis. His health up to that time had been good. The chill seized him at two o'clock in the night, and continued till day, when sinapisms, hot teas, and hot bricks applied to the extremities induced a partial reaction. Violent pain in the eyes, and across the forehead, succeeded, attended with frequent vomitings of a tough mucus, tinged with bile and blood.

Seventh. I was called to visit the patient about eleven o'clock, A. M.; found him complaining of a deep seated pain in the head and eyes, sense of burning in the breast, thirst, nausea, accompanied by vomiting; skin moist and cool, great prostration, pulse small, quick, tremulous; cough, with hurried and laborious breathing; eyes red and half closed; edges of the tongue white, a dark stripe in the middle, coated with a tenacious mucus and bile; low muttering delirium, and great alarm.

*Treatment.*—A gentle cathartic, with anodynes and stimulants, and sinapisms to the extremities.

Two o'clock, P. M. Expectoration freer, mucus tinged with blood, skin has regained its natural warmth, pulse of better volume, and more regular beat; mind clearer, but the patient is very fearful of dying; has taken no nourishment; great tossing.

Eighth. Vomits a chocolate colored matter; breath very offensive; pulse more tremulous; mental wanderings; bowels have been moved gently, passages natural: same treatment to be continued.

Ninth. Considered by his attendants to be better: took some nourishment about day light, turned on his right side, and expired unobserved.

*Autopsy.*—Heart much larger than natural; stomach natural in color, mucous membrane softened; spleen not more than half the natural size; liver much enlarged, surface paler than natural, substance paler, and softened, vessels engorged with blood; gall-bladder filled with a fluid resembling a mixture of blood and bile. Lungs. The left lobe presented nothing abnormal. Of the right lung, the only parts free from disease were the anterior portions of the superior and middle lobes, the whole of the remaining portions were completely hepatized. The brain was not examined.

The epidemic declined with the opening of the spring, and I am happy to be able to state that our village and county are now free from it, and perfectly healthy.

June 9th, 1843.

